# Clinical characteristics of new-onset acute kidney injury in patients with established acute respiratory distress syndrome: A prospective single-center *post hoc* observational study

**DOI:** 10.3389/fmed.2022.987437

**Published:** 2022-09-20

**Authors:** Xiaoyang Cui, Xu Huang, Xin Yu, Ying Cai, Ye Tian, Qingyuan Zhan

**Affiliations:** Department of Pulmonary and Critical Care Medicine, Center of Respiratory Medicine, National Clinical Research Center for Respiratory Diseases, Institute of Respiratory Medicine, China-Japan Friendship Hospital, Beijing, China

**Keywords:** acute kidney injury, acute respiratory distress syndrome, extra-pulmonary complications, mechanical ventilation, fluid balance, nephrotoxic agents

## Abstract

**Background:**

We assessed the incidence and clinical characteristics of acute kidney injury (AKI) in acute respiratory distress syndrome (ARDS) patients and its effect on clinical outcomes.

**Methods:**

We conducted a single-center prospective longitudinal study. Patients who met the Berlin definition of ARDS in the medical ICU in China-Japan Friendship Hospital from March 1, 2016, to September 30, 2020, were included. AKI was defined according to the KDIGO clinical practice guidelines. Early and late AKI were defined as AKI occurring within 48 h after ARDS was diagnosed or after 48 h, respectively.

**Results:**

Of the 311 ARDS patients, 161 (51.8%) developed AKI after ICU admission. Independent risk factors for AKI in ARDS patients were age (OR 1.027, 95% CI 1.009–1.045), a history of diabetes mellitus (OR 2.110, 95%CI 1.100–4.046) and chronic kidney disease (CKD) (OR 9.328, 95%CI 2.393–36.363), APACHE II score (OR 1.049, 95%CI 1.008–1.092), average lactate level in the first 3 days (OR 1.965, 95%CI 1.287–3.020) and using ECMO support (OR 2.359, 95%CI 1.154–4.824). Early AKI was found in 91 (56.5%) patients and late AKI was found in 70 (43.5%). Early AKI was related to the patient’s underlying disease and the severity of hospital admission, while late AKI was related to the application of nephrotoxic drugs. The mortality rate of ARDS combined with AKI was 57.1%, which was independently associated with shock (OR 54.943, 95%CI 9.751–309.573).

**Conclusion:**

A significant number of patients with ARDS developed AKI, and the mortality rate for ARDS patients was significantly higher when combined with AKI. Therapeutic drug monitoring should be routinely used to avoid drug toxicity during treatment.

## Background

Acute respiratory distress syndrome (ARDS) is a syndrome consisting of acute hypoxemic respiratory failure onset within 1 week of insult or the presence of new (within 7 days) or worsening respiratory symptoms with bilateral pulmonary infiltrates that is associated with both pulmonary and non-pulmonary risk factors and is not primarily due to cardiac failure and fluid overload (1). It has a substantial impact on public health, with an incidence of 86.2 per 100,000 person-years and an in-hospital mortality rate of 38.5% (2). Patients with ARDS may have a severe inflammatory response due to ARDS or its treatments, which may cause extrapulmonary organ dysfunction and contribute to death (3–6). Acute kidney injury (AKI) is a common, serious complication in critically ill patients that may result in increased mortality, longer hospital stays and higher medical costs (7, 8). Previous studies have shown that ARDS is independently associated with AKI (9). Experimental studies have shown that one mechanism of renal injury in ARDS is positive-pressure ventilation that can result in inadequate renal perfusion possibly through a reduced cardiac output and increased central venous pressure (10). In addition, hypoxemia, hypercarbia, and systemic acidosis could influence renal vascular resistance and renal perfusion pressures, resulting in AKI. Moreover, respiratory support methods such as mechanical ventilation, extracorporeal membrane oxygenation (ECMO) and ventilator-induced lung injury can activate systemic inflammation via the release of inflammatory cytokines, which may cause renal injury (11–13). Multicenter studies show that the mortality rate for ARDS in China is 68.5% in adult ICUs, and when combined with AKI, the mortality rate is as high as 80% (14). To date, few studies have comprehensively discussed the effects of ARDS on patients’ renal function based on patient status, ARDS diagnosis, and specific interventions performed in the ICU.

The primary objective of this study was to assess the incidence and characteristics of AKI in ARDS patients and to study the risk factors associated with its development. The secondary objective was to identify the 28-day in-hospital mortality rate of AKI in ARDS patients and the variables associated with mortality.

## Materials and methods

### Study design and data source

**FIGURE 1:**
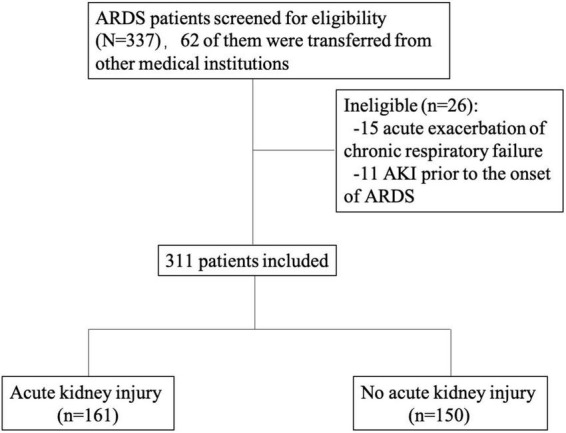
Consort flow chart.

This was a single-center prospective longitudinal study which is a part of a registered study (Clinicaltrials.gov NCT02975908). We consecutively included all adult patients (>18 years old) who met the ARDS Berlin definition for the first incidence of ARDS in the medical ICU in China-Japan Friendship Hospital from March 1, 2016, to September 30, 2020 (1). The exclusion criteria were as follows: chronic respiratory failure due to chronic respiratory diseases, such as chronic obstructive pulmonary disease; bronchiectasis or lung fibrosis; AKI prior to the onset of ARDS; and inability or unwillingness to provide informed consent (Figure 1). The study protocol was approved by the China-Japan Friendship Hospital ethics committee. China-Japan Friendship Hospital is the national clinical research center for respiratory disease in China, so most ARDS patients in the MICU are intrapulmonary ARDS. The respiratory support our center can provide includes High-flow Nasal Cannula (HFNC), non-invasive positive pressure ventilation (NPPV), invasive positive pressure ventilation (IPPV) and extracorporeal membrane oxygenation (ECMO) as well as continuous renal replacement therapy (CRRT) renal support. The clinician decided the methods of ventilation and when to start the renal support according to the guidelines (15, 16). Epidemiological, demographic, clinical, laboratory, treatment and outcome data were extracted from electronic medical records using the specialized case report form by the investigators. The data for this study were a *post hoc* analysis derived from this registry database.

### Definitions

The clinical diagnosis of ARDS, according to the Berlin ARDS definition, includes the following: (1) the presence of acute hypoxemic respiratory failure; (2) onset of respiratory symptoms within 1 week or the presence of new (within 7 days) or worsening respiratory symptoms; (3) bilateral opacities on chest X-ray or computed tomography not fully explained by effusions, lobar or lung collapse, or nodules; and (4) acute hypoxemic respiratory failure not caused by cardiac failure or fluid overload. All ICU patients who had acute hypoxemia with PaO_2_/FIO_2_ (P/F) ≤ 300 mmHg were screened for ARDS. Patients with ARDS were categorized on the day of ARDS diagnosis based on their PaO2/FIO2 ratios into mild (200 < PaO_2_/FIO_2_ ≤ 300 mmHg), moderate (100 < PaO2/FIO2 ≤ 200 mmHg), and severe (PaO2/FIO2 ≤ 100 mmHg) according to the Berlin definition. AKI was diagnosed and graded according to the Kidney Disease Improving Global Outcomes (KDIGO) 2012 guidelines using serum creatinine (SCr) and urine output criteria (16). Patient baseline SCr values were obtained between 7 and 365 days before hospitalization. However, if the measured baseline SCr was unavailable, an estimated baseline creatinine, according to the Modification of Diet in Renal Disease (MDRD) formula assuming a GFR of 75 ml/min, was used (17, 18). If a patient had a reported history of CKD, their estimated SCr was back-calculated with the MDRD formula assuming a GFR of 60 ml/min. Renal recovery was defined as alive, free of renal replacement treatment (RRT), improvement in SCr < 1.5 times the baseline SCr, and urine output > 0.5 mL/kg/h for more than 6 h (19). Early AKI was defined as AKI occurring within 48 h of ARDS diagnosis. Patients who developed AKI later than 48 h after ARDS diagnosis were classified into the late AKI group (20). The observation period of this study was from admission to discharge.

Day 1 was defined as the first day that the ARDS criteria were met after ICU admission. Each patient with ARDS was followed up to 28 days after diagnosis, until hospital discharge, or death. The risk factors for ARDS included intrapulmonary (pneumonia, aspiration, and drowning pulmonary contusion) and extrapulmonary factors (trauma, extrapulmonary sepsis, non-cardiogenic shock and blood transfusion) (21). The acute physiology and chronic health evaluation (APACHE) II score and sequential organ failure assessment (SOFA) score were recorded using data from the first 24 h in the ICU. SOFA scores ≥ 3 referring to one organ were defined as failure of the respective organ. Shock was defined as arterial systolic blood pressure less than 90 mmHg, mean arterial pressure less than 60 mmHg, or the use of norepinephrine at any dosage to maintain systolic blood pressure at 90 mmHg or higher or mean arterial pressure at 60 mmHg or higher. Barotrauma refers to pneumothorax, pneumomediastinum, subcutaneous emphysema, pneumopericardium. Invasive ventilator-free days were calculated as the number of days from weaning from invasive ventilation to day 28. Patients who died before weaning were considered to have a ventilator-free-day value of 0. The immunosuppressed state included the following: corticosteroid or immunosuppressant use within 90 days, seropositive for human immunodeficiency virus, malignant tumor, and patients receiving radiation therapy or chemotherapy for an underlying malignancy within 90 days (22). The respiratory support method recorded in this paper is the maximal respiratory support method for the patient during hospitalization. Mechanical ventilation parameters, arterial blood gas, and fluid balance were collected for the first 14 days and on Day 21 and Day 28 after the onset of ARDS. Laboratory examination (blood examinations, coagulation profiles and BNP or NT-proBNP) was recorded for the first 4 days and on Day 7. Serum creatinine, urine volume, and the use of RRT were recorded daily until 28 days after ARDS diagnosis or hospital discharge to determine the highest stage of AKI. Fluid input included all fluids infused by intravenous or enteral routes. Fluid output included urine output, volume of fecal matter and fluid loss from drains. Fluid balance was calculated as the difference between fluid input and fluid output. All data were recorded as close as possible to 8 AM each day. Exposure to nephrotoxic agents included vancomycin, aminoglycoside, sulfamethoxazole-trimethoprim, colistin, amphotericin B, voriconazole, angiotensin converting enzyme inhibitors/angiotensin receptor blockers (ACEIs/ARBs), diuretics, cyclosporine and non-steroid anti-inflammatory drugs (NSAIDs) before AKI was diagnosed.

### Statistical analysis

Categorical variables were analyzed using the χ^2^ test or Fisher’s exact test, and continuous variables were analyzed using Student’s *t*-test or the Mann–Whitney *U* test. After testing the distribution of continuous variables, normally distributed variables were presented as the mean ± SD, and non-normally distributed variables were presented as the median (interquartile range). Multivariate analysis was performed to evaluate potential explanatory variables (i.e., variables found to be both clinically significant and statistically significant in univariate analyses) for AKI and clinical outcomes, which were expressed as the odds ratio (OR) and its 95% confidence interval (CI). Survival was assessed by the Kaplan–Meier method and compared between groups using the log-rank test. The hazard ratios of variables related to 28-day mortality were estimated with a Cox proportional hazards regression model. A two-sided p value less than 0.05 was considered statistically significant. SPSS 24.0 (SPSS, Chicago, IL, USA) was used for all statistical analyses (22).

## Results

### Baseline characteristics and clinical features

During the observation period, a total of 311 patients were diagnosed with ARDS and met the inclusion criteria. Of these patients, the mean age was 56.6 years, and 216 (69.5%) were male. One hundred twenty-two (39.2%) patients had a history of hypertension, 68 (21.9%) had diabetes mellitus, 32 (10.3%) had coronary heart disease, 26 (8.4%) had CKD, 23 (7.4%) had chronic respiratory disease, 3 (0.9%) had chronic liver disease, 28 (9%) had cerebrovascular disease, and 27 (8.7%) had connective tissue disease. Seven (2.3%) patients had a history of drinking, 124 (39.9%) patients had a history of smoking, 10 (3.2%) patients had surgery in the past 3 months, and 124 (39.9%) patients were immunosuppressed. AKI developed in 161 (51.8%) of the 311 patients with ARDS: 91 (56.5%) patients developed AKI within 48 h of ARDS diagnosis, whereas 70 (43.5%) developed AKI later than 48 h. Basal creatinine values were available for 28 patients, and the remaining 283 patient’s baseline creatinine were estimated by back calculation with the MDRD equation. There were no missing results for other parameters in the result section and also no lost-to-follow-up populations in this study.

### Factors and clinical outcomes associated with the development of acute kidney injury

Patients in the AKI group were significantly older (59.7 ± 16.5 vs. 53.2 ± 15.8 years, *p* = 0.00) and had a greater proportion of hypertension (44.7% vs. 33.3%, *p* = 0.04), diabetes mellitus (27.3% vs. 16%, p = 0.02), coronary heart disease (13.7% vs. 6.7%, *p* = 0.04) and CKD (14.3% vs. 2%, *p* = 0.00) than patients in the non-AKI group. Patients with AKI had a greater proportion of multiple comorbidities (Table 1). Table 2 shows the physiology and organ dysfunction parameters on admission, laboratory results and treatments. Admission APACHE II (21 vs. 16, *p* = 0.00) and SOFA scores (8 vs. 5, *p* = 0.00) were significantly higher in patients with AKI than in those without AKI. Shock was observed in 109 (67.7%) of 161 patients with AKI and in 48 (32%) of 150 patients without AKI (*p* = 0.00). There was no significant difference in the cause or severity of ARDS between the two groups. Regarding the laboratory indicators, patients with AKI had a higher prevalence of metabolic acidosis in the first 3 days and a higher level of lactic acid. In addition, AKI patients’ white blood cell and BNP/NT-proBNP levels were significantly higher than those of patients without AKI. However, there was no association between ventilator parameter settings, airway plateau pressure, peak airway pressure, airway resistance, compliance and the development of AKI among ARDS patients. The number of patients in the AKI group who needed NPPV was significantly lower than that in the non-AKI group (12 vs. 24, *p* = 0.02), whereas the number of patients who needed ECMO support in the AKI group was significantly higher than that in the non-AKI group (41 vs. 21, *p* = 0.01). AKI patients had more fluid accumulation than non-AKI patients on Day 1 (285 vs. 0, *p* = 0.00) and Day 6 (153 vs. –32, *p* = 0.03).

**TABLE 1:** Baseline characteristics by acute kidney injury.

	AKI (*n* = 161)	Non-AKI (*n* = 150)	*p*
Age, mean(SD), years	59.7 (16.5)	53.2 (15.8)	0.00^a^
Male, (*n*%)	119 (73.9%)	97 (64.7%)	0.08
BMI, median(IQR), kg/m^2^	24(22,27)	24(22,27)	0.22
**Comorbidities, (*n*%)**			
Hypertension	72 (44.7%)	50 (33.3%)	0.04^a^
Diabetes mellitus	44 (27.3%)	24 (16%)	0.02^a^
Coronary heart disease	22 (13.7%)	10 (6.7%)	0.04^a^
Congestive heart failure	8 (5%)	2 (1.3%)	0.07
Chronic kidney disease	23 (14.3%)	3 (2%)	0.00^a^
Chronic respiratory disease	11 (6.8%)	12 (8%)	0.69
Chronic liver disease	1 (0.6%)	2 (1.3%)	0.52
Cerebrovascular disease	19 (11.8%)	9 (6%)	0.05
Connective tissue disease	17 (10.6%)	10 (6.7%)	0.22
Alcohol drinking	3 (1.9%)	4 (2.7%)	0.63
0 comorbidity	40(24.8%)	44(29.5%)	0.37
1 comorbidity	26 (16.1%)	48 (32%)	0.00^a^
2 comorbidities	48 (29.8%)	37 (24.7%)	0.37
2 + comorbidities	47 (29.2%)	21 (14%)	0.00^a^
Smoking	72 (44.7%)	52 (34.7%)	0.07
Surgery in the past 3 months	3 (1.9%)	7 (4.7%)	0.16
Immunosuppression	64 (39.8%)	60 (40%)	0.96
Transferred from other hospital	34(21.1%)	28(18.8%)	0.14
**Cause of ARDS, (*n*%)**			
Intrapulmonary	154 (95.7%)	143 (95.3%)	0.89

AKI, acute kidney injury; ARDS, acute respiratory distress syndrome; BMI, body mass index; SD, standard deviation; IQR, interquartile range.

^a^p < 0.05.

**TABLE 2:** Physiology and organ dysfunction parameters on admission, ventilator settings on day 1, arterial blood gas on day 1–3, laboratory indicators, medication history and fluid balance.

	AKI (161)	Non-AKI (150)	*p*
APACHE II, median (IQR)	21 (16, 26)	16 (11, 22)	0.00^a^
SOFA, median (IQR)	8 (6, 12)	5 (3, 8)	0.00^a^
**Severity of ARDS on day 1, (*n*%)**			
Mild	21 (13%)	22 (14.7%)	0.68
Moderate	84 (52.2%)	72 (48%)	0.46
Severe	56 (34.8%)	56 (37.3%)	0.64
Shock, (*n*%)	109 (67.7%)	48 (32%)	0.00^a^
**Arterial blood gases (averaged on day 1–3)**			
pH, median (IQR)	7.41 (7.36,7.45)	7.45 (7.4,7.47)	0.00^a^
PaCO2, median (IQR), mmHg	38.2 (32.4,45.6)	36 (32,42.5)	0.10
PaO2, median (IQR), mmHg	81.3 (70.7, 92.8)	80.33 (68.8,91.2)	0.34
PaO2/FiO2, median (IQR)	123.7 (93.7, 170.7)	133 (96.5,185.9)	0.29
HCO3-, mean (*SD*), mmol/L	23.5 (4.2)	24.6 (3.9)	0.02^a^
cLac, median (IQR), mmol/L	1.7 (1.3,2.1)	1.4 (1.2,1.7)	0.00^a^
Ventilator settings			
Vt/kg, median (IQR), ml/kg	6.2(5.1, 8.0)	6.4(4.6, 8.0)	0.99
PEEP, median (IQR), cmH_2_O	9 (7, 12)	10 (7.5, 12)	0.55
FiO_2_, median (IQR)	0.7(0.5, 0.8)	0.7 (0.5, 0.8)	0.63
Pplat, mean (*SD*), cmH_2_O	20.6 (6.2)	18.3 (6.2)	0.49
Ppeak, mean (*SD*), cmH_2_O	24 (5)	24.6 (4.6)	0.42
C, mean (*SD*), ml/cmH_2_O	39.5 (19.8)	34.6 (14)	0.62
R, median (IQR), cmH_2_O/L/S	15.4 (12.8, 18.7)	13 (10, 17.5)	0.23
**Laboratory examination, median (IQR)**			
WBC, × 109/L	10.9 (6.8, 16.3)	8.6 (5.7, 13.1)	0.02^a^
NEU, × 109/L	8.6 (4.1, 13.5)	7.4 (4.4, 11.3)	0.26
LYM, × 109/L	0.5 (0.3, 1)	0.5 (0.3, 0.9)	0.69
BNP, pg/ml	244 (88.2, 602.9)	101 (39, 251.3)	0.00^a^
NT-proBNP, pg/ml	1414 (5145.3,380.3)	369 (135.5,1075.5)	0.00^a^
**Maximal respiratory support, (*n*%)**			
HFNC	20 (12.4%)	30 (20%)	0.07
NPPV	12 (7.4%)	24 (16%)	0.02^a^
IPPV	88 (54.7%)	75 (50%)	0.41
ECMO	41 (25.5%)	21 (14%)	0.01^a^
Barotrauma, (*n*%)	8	0	
**Nephrotoxic agents, (*n*%)**			
Vancomycin	17 (10.6%)	27 (18%)	0.06
Aminoglycoside	15 (9.3%)	13 (8.7%)	0.84
Sulfamethoxazole- Trimethoprim	51 (31.7%)	49 (32.7%)	0.85
Colistin	13 (8.1%)	14 (9.3%)	0.69
Amphotericin B	19 (11.8%)	11 (7.3%)	0.18
ACEI/ARB	1 (0.6%)	1 (0.7%)	0.96
NSAIDs	15 (9.3%)	19 (12.7%)	0.34
Diuretics	48 (29.8%)	58 (38.7%)	0.10
Voriconazole	35 (21.7%)	40 (26.7%)	0.31
Cyclosporine	4 (2.5%)	4 (2.7%)	0.92
**Fluid balance, median (IQR), ml**			
D1	285 (–242,987)	0 (–492,561)	0.00^a^
D2	365 (–496,1169)	179 (–445,869)	0.28
D3	–131 (–987,773)	54 (–749,737)	0.48
D4	–219 (–976,915)	–20 (–641,604)	0.92
D5	16 (–746,1010)	–180 (–740,436)	0.15
D6	153 (–545,919)	–32 (–1028,473)	0.03^a^
D7	–20 (–619,796)	–95 (–792,600)	0.15
CRRT, (*n*%)	38 (23.6%)	7 (4.6%)	0.00^a^
Onset of CRRT, days, median(IQR)	1(0,2.3)	3(1,6)	0.06

APACHE, acute physiology, age, chronic health evaluation; SOFA, sequential organ failure assessment; VT, tidal volume; PEEP, positive end- expiratory pressure; Pplat, plateau pressure values; Ppeak, peak inspiratory pressure; C, compliance; R, resistance; HFNC, high flow nasal cannula; IPPV, invasive positive pressure ventilation; NPPV, non- invasive positive pressure ventilation; EMCO, extracorporeal membrane oxygenation; ACEI, angiotensin-converting enzyme inhibitors; ARB, angiotensin receptor blocker; NSAIDs, non-steroidal antiinflammatory drugs; CRRT, continuous renal replacement therapy.

^a^p < 0.05.

After adjustment in a multivariable analysis model, the development of AKI was independently associated with age [OR 1.027, 95% CI 1.009–1.045], a history of diabetes mellitus [OR 2.110, 95% CI 1.100–4.046], a history of CKD [OR 9.328, 95% CI 2.393–36.363], APACHE II score [OR 1.049, 95% CI 1.008–1.092], the average lactate level in the first 3 days [OR 1.965, 95% CI 1.287–3.020] and the use of ECMO support [OR 2.359, 95% CI 1.154–4.824].

**FIGURE 2:**
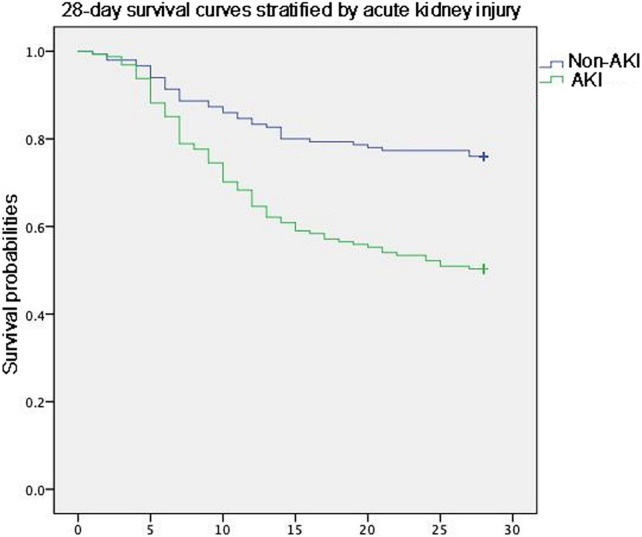
Kaplan–Meier analysis revealed a significantly higher 28-day mortality rate for patients with AKI.

Although the length of ICU stay did not differ between the patients with ARDS with and without AKI, the invasive ventilation-free days to Day 28 were significantly shorter. Moreover, the 28 days mortality and ICU mortality were apparently higher in patients with AKI (Table 3). Kaplan–Meier analysis revealed a significantly higher 28-day mortality rate for patients with AKI (Log-Rank chi-square = 21.313, *p* = 0.00, Figure 2). The hazard ratio for AKI in 28-day mortality of ARDS patients, estimated using a Cox proportional hazards regression model, was 1.488 (95% CI 1.006–2.201, *p* = 0.046).

**TABLE 3:** Outcomes among patients with and without acute kidney injury.

	AKI (*n* = 161)	Non-AKI (*n* = 150)	*p*
Invasive ventilation-free days to day 28, mean (*SD*)	8.4 (10.8%)	16.5 (11.9%)	0.00^a^
Length of ICU stay, mean (*SD*)	22.6 (21.2%)	22.1 (23.6%)	0.84
Withdrawal of treatment, (*n*%)	19(11.8%)	15(10.0%)	0.72
28 days mortality, (*n*%)	80(49.7%)	36(24.0%)	0.00^a^
ICU Mortality, (*n*%)	92 (57.1%)	40 (26.7%)	0.00^a^

^a^p < 0.05.

Acute kidney injury was significantly more prevalent in patients with ECMO support. But the onset of AKI, CRRT and 28-day mortality was no different in ECMO patients (Table 4).

**TABLE 4:** The onset of AKI, CRRT and 28-day mortality of ARDS patients with and without ECMO support.

	ECMO (*n* = 62)	Non-ECMO (*n* = 249)	*p*
AKI, (*n*%)	41(66.1%)	120(48.2%)	0.01^a^
onset of AKI, days, median (IQR)	1(1, 3)	1(0, 5)	0.64
onset of CRRT, days, median (IQR)	1(0, 5)	1.5(0, 3)	0.94
28-day Mortality, (*n*%)	25(40.3%)	91(36.5%)	0.56

^a^p < 0.05.

### Factors associated with the in-hospital mortality of patients with acute respiratory distress syndrome with acute kidney injury

Overall, the in-hospital mortality rate for ARDS patients was 42.4%. However, the mortality rate was 57.1% in patients with ARDS with AKI. In univariate analysis, the patients who died with ARDS and AKI were older (62.9 vs. 55.5, *p* = 0.00), had a lower body mass index (23.9 vs. 25.4, *p* = 0.03) and were less likely to be hypertensive (35.9% vs. 56.9%, *p* = 0.01). The incidence of shock was significantly higher among deceased patients (93.5% vs. 33.3%, *p* = 0.00). Mild ARDS was more common in survivors (21.7% vs. 6.5%, *p* = 0.01). The P_*a*_O_2_/FiO_2_ was higher (143 vs. 112.9, *p* = 0.00) and the FiO_2_ (0.6 vs. 0.7, *p* = 0.00) was lower in this group. Due to the higher shock incidence, a lower PaO_2_/FiO_2_ and more severe ARDS status, the deceased patients had significantly higher cLac levels (1.8 vs. 1.6, *p* = 0.00). For AKI severity, the majority of deceased patients were in stage 2, whereas most survivors were in stage 3, and the baseline Cr was significantly higher in the survivor group (143.3 vs. 86.6, *p* = 0.00). Ventilator-specific variables and nephrotoxic drugs were not correlated with the mortality of ARDS combined with AKI patients. However, regarding fluid accumulation, the deceased patients had significantly greater fluid accumulation than the survivors, especially on D2, D3, D4, and D7 (Table 5).

**TABLE 5:** Factors associated with the in-hospital mortality of ARDS patients combined with AKI.

	Survivors (*n* = 69)	Unsurvivors (*n* = 92)	*p*
**Baseline characteristics**			
Age, mean (*SD*), years	55.5 (15.3)	62.9 (16.8)	0.00^a^
Male, (*n*%)	48 (69.6%)	71 (77.2%)	0.28
BMI, mean (*SD*)	25.4 (4.2)	23.9 (4.2)	0.03^a^
**Comorbidities, (*n*%)**			
Hypertension	39 (56.9%)	33 (35.9%)	0.01^a^
Diabetes mellitus	22 (31.9%)	22 (23.9%)	0.26
Coronary heart disease	10 (14.5%)	12 (13%)	0.79
Congestive heart failure	4 (5.8%)	4 (4.3%)	0.68
Chronic kidney disease	12 (17.4%)	11 (12%)	0.33
Chronic respiratory disease	3 (4.3%)	8 (8.7%)	0.28
Chronic liver disease	0 (0%)	1 (1.1%)	
Cerebrovascular disease	12 (17.4%)	7 (7.6%)	0.06
Connective tissue disease	7 (10.1%)	10 (10.9%)	0.88
Alcohol drinking	1 (1.4%)	2 (2.2%)	1.00
Smoking	31 (44.9%)	41 (45.1%)	0.99
Immunosuppression	27 (39.1%)	37 (40.2%)	0.89
APACHE II, mean (*SD*)	21.4 (6.8)	22.1 (7.9)	0.52
SOFA, mean (*SD*)	8.8 (4)	8.9 (4.4)	0.84
Shock, (*n*%)	23 (33.3%)	86 (93.5%)	0.00^a^
**Cause of ARDS, (*n*%)**			
Intrapulmonary	64 (92.8%)	90 (97.8%)	0.14
**Severity of ARDS on day 1, (*n*%)**			
Mild	15 (21.7%)	6 (6.5%)	0.01^a^
Moderate	35 (50.7%)	51 (55.4%)	0.55
Severe	19 (27.5%)	35 (38.0%)	0.16
**Arterial blood gas analysis (average on day 1–3)**			
pH, median (IQR)	7.41 (7.37,7.45)	7.41 (7.34,7.45)	0.50
PaCO2, median (IQR), mmHg	37.2 (32,43.1)	39.3 (32.7,47.7)	0.18
PaO2, median (IQR), mmHg	83.7 (72.6,93.7)	79.8 (69.4,92)	0.32
P_*a*_O_2_/FiO_2_, median (IQR)	143 (112.8,207)	112.9 (86.9,148.1)	0.00^a^
HCO3-, mean (SD), mmol/L	23.5 (4.2)	23.5 (4.2)	0.95
cLac, median (IQR), mmol/L	1.6 (1.2,2.1)	1.8 (1.5,2.3)	0.00^a^
**Ventilator settings, mean (*SD*)**			
Vt/Kg, ml	6.8 (1.7)	6.9(1.8)	0.72
PEEP, cmH2O	10 (4.4)	9.1 (2.8)	0.39
FiO2	0.6 (0.2)	0.7 (0.2)	0.00^a^
Pplat, cmH2O	19.3 (5.5)	21.6 (6.7)	0.44
Ppeak, cmH2O	23.7 (5.1)	24.2 (5)	0.64
C, ml/cmH2O	32.7 (16.9)	43.5 (20.8)	0.20
R, cmH2O/L/S	17.9 (5.9)	15.5 (7.5)	0.43
**Laboratory examination, mean (*SD*)**			
WBC, × 109/L	11.1 (6.4)	14 (12)	0.09
NEU, × 109/L	8.6 (6.4)	11.2 (10.5)	0.08
LYM, × 109/L	0.7 (0.6)	0.8 (1.2)	0.84
BNP, pg/ml	385.1 (431.5)	621.2 (1027.2)	0.19
NT-proBNP, pg/ml	2295.6 (2963.7)	5462.2 (8662.5)	0.08
Baseline Cr, median (IQR), mmol/L	143.3 (83.9,285.2)	86.6 (67.6,128.8)	0.00^a^
Peak serum creatinine, median (IQR), mmol/L	229.1 (151.4,593)	238.6 (169.7, 319.9)	0.41
Serum creatinine increase, median (IQR), mmol/L	141.4 (60.7, 485.1)	147.2 (81.6, 224.7)	0.39
**Maximal respiratory support (*n*%)**			
HFNC	5 (7.2%)	15 (16.3%)	0.09
NPPV	3 (4.3%)	9 (9.8%)	0.19
IPPV	41 (59.4%)	47 (51.1%)	0.29
ECMO	20 (29.0%)	21 (22.8%)	0.38
Baro-trauma	4 (5.8%)	4 (4.3%)	0.73
**Severity of AKI (*n*%)**			
Stage 1	21 (30.4%)	20 (21.7%)	0.21
Stage 2	13 (18.8%)	37 (40.2%)	0.00^a^
Stage 3	35 (50.7%)	35 (38%)	0.11
**Nephrotoxic drug (*n*%)**			
Vancomycin	7 (10.1%)	10 (10.9%)	0.06
Aminoglycoside	3 (4.3%)	12 (13%)	0.06
Sulfamethoxazole- trimethoprim	17 (24.6%)	34 (37%)	0.09
Colistin	3 (4.3%)	10 (10.9%)	0.13
Amphotericin B	6 (8.7%)	13 (14.1%)	0.29
ACEI/ARB	1 (1.1%)	0 (0%)	1.00
NSAIDs	6 (8.7%)	9 (9.8%)	0.81
Diuretics	19 (27.5%)	29 (31.5%)	0.58
Voriconazole	12 (17.4%)	23 (25%)	0.25
Cyclosporine	3 (4.3%)	1 (1.1%)	0.19
**Fluid balance, ml**			
D1, median (IQR)	285 (–386.5,96)	285 (–117.8,1068.8)	0.27
D2, mean (*SD*)	–122.2 (1365.9)	778.5 (1508.7)	0.00^a^
D3, median (IQR)	–453 (–1374,247)	319.5 (–795,1227)	0.00^a^
D4, mean (*SD*)	–416.7 (1364.5)	311.8 (1812.1)	0.00^a^
D5, mean (*SD*)	–122.9 (1303.5)	143.3 (2123.9)	0.38
D6, mean (*SD*)	–65.2 (1335.3)	364.4 (1570.8)	0.09
D7, mean (*SD*)	–188.6 (1413)	428.4 (1414.1)	0.02^a^
CRRT, (*n*%)	15 (21.7%)	23 (25%)	0.63
Onset of CRRT, days, median (IQR)	1 (0,2)	1(0,3)	0.57

^a^p < 0.05.

After adjustment in a multivariable analysis model, the mortality of patients with ARDS combined with AKI was independently associated with shock (OR 54.943, 95% CI 9.751–309.573).

### Factors associated with the development of early and late acute kidney injury

Early AKI was found in 91 (56.5%) patients, and late AKI was found in 70 (43.5%) patients. Patients in the early AKI group had more hypertension (54.9% vs. 31.4%, *p* = 0.00) and CKD (24.2% vs. 1.4%, *p* = 0.00) than those in the late AKI group. Compared with patients in the late AKI group, patients in the early AKI group were more severely ill when they were admitted to the ICU based on the higher APACHE II and SOFA scores. In addition, patients in the early AKI group had a higher prevalence of metabolic acidosis and higher BNP/NT-proBNP levels. Regarding the stage of AKI between the two groups, the majority of patients in the early AKI group were in stage 3 (*n* = 47, 51.6%), whereas half of the patients in the late AKI group were in stage 1 and stage 2 (*n* = 47, 67.1%). Therefore, the peak serum creatinine in the early-onset group was significantly higher than that in the late-onset group (259.8 vs. 197.3, *p* = 0.00), and the use of CRRT was significantly higher in the early-onset group (30.8% vs. 14.3%, *p* = 0.02). The use of nephrotoxic agents, especially aminoglycosides (17.1% vs. 3.3%, *p* = 0.01), colistin (17.1% vs. 1.1%, *p* = 0.00), amphotericin B (20% vs. 5.5%, *p* = 0.01), NSAIDs (17.1% vs. 3.3%, *p* = 0.01), diuretics (44.3% vs. 18.7%, *p* = 0.00) and voriconazole (34.3% vs. 12.1%, *p* = 0.00), was significantly associated with the development of late-onset AKI. The fluid accumulation on Day 7 was significantly higher in the late-onset group than in the early-onset group. Regarding the prognostic indicators, the number of invasive ventilation-free days up to Day 28 in the early-onset group was significantly longer than that in the late-onset group, and the mortality rate was significantly lower in the early-onset group (Table 6).

**TABLE 6:** Factors associated with the development of early and late AKI.

	Early AKI (*n* = 91)	Late AKI (*n* = 70)	*p*
**Baseline characteristics**			
Age, mean, (years)	60.2 (16.6)	59 (16.6)	0.65
Male, (*n*%)	64 (70.3%)	55 (78.6%)	0.24
BMI	24.7 (4.2)	24.3 (4.4)	0.59
**Comorbidities, (*n*%)**			
Hypertension	50 (54.9%)	22 (31.4%)	0.00^a^
Diabetes mellitus	28 (30.8%)	16 (22.9%)	0.26
Coronary heart disease	13 (14.3%)	9 (12.9%)	0.79
Congestive heart failure	7 (7.7%)	1 (1.4%)	0.14
Chronic kidney disease	22 (24.2%)	1 (1.4%)	0.00^a^
Chronic respiratory disease	5 (5.5%)	6 (8.6%)	0.44
Chronic liver disease	0 (0%)	1 (1.4%)	0.44
Cerebrovascular disease	11 (12.1%)	8 (11.4%)	0.89
Connective tissue disease	11 (12.1%)	6 (8.6%)	0.47
Alcohol drinking	2 (2.2%)	1 (1.4%)	1.00
Smoking	35 (38.5%)	37 (52.9%)	0.07
Immunosuppression	40 (44%)	24 (34.3%)	0.21
APACHE II, mean (*SD*)	24.2(7.5)	18.6 (6.3)	0.00^a^
SOFA, mean (*SD*)	10.2 (4.2)	7 (3.6)	0.00^a^
Shock	58 (63.7%)	51 (72.9%)	0.22
**Cause of ARDS, (*n*%)**			
Intrapulmonary	84 (92.3%)	70 (100%)	0.02^a^
Severity of ARDS, (*n*%)			
Mild	13 (14.3%)	8 (11.4%)	0.59
Moderate	45 (49.5%)	39 (55.7%)	0.43
Severe	33 (36.3%)	23 (32.9%)	0.65
**Arterial blood gas analysis (average on day 1–3)**			
pH, median (IQR)	7.39 (7.34,7.44)	7.43 (7.37,7.46)	0.00^a^
PaCO2, median (IQR), mmHg	38.5 (32.3,45.1)	38.2 (32.6,46)	0.82
PaO2, median (IQR), mmHg	84.2 (72.6,94.5)	79.3 (70.3,90.5)	0.08
PaO2/FiO2, median (IQR)	126.7 (100,179)	120.3 (88,159.2)	0.16
HCO3-, mean (*SD*), mmol/L	22.7 (3.3)	24.6 (4)	0.00^a^
cLac, median (IQR), mmol/L	1.8 (1.3,2.5)	1.7 (1.3,2)	0.30
**Ventilator settings**			
Vt, median (IQR), ml	454.5 (118.2)	429.6 (114.3)	0.34
PEEP, median (IQR), cmH2O	9.6 (3.6)	9.6 (3.7)	0.99
FiO2, median (IQR)	0.7 (0.2)	0.7 (0.2)	0.32
Pplat, mean (*SD*), cmH2O	20 (6.3)	22.2 (6.5)	0.51
Ppeak, mean (*SD*), cmH2O	24.3 (5.5)	23.6 (4.3)	0.46
**Laboratory examination**			
WBC, × 109/L	13.5 (11.6)	11.7 (7.4)	0.28
NEU, × 109/L	10.6 (10.2)	9.4 (7.4)	0.41
LYM, × 109/L	0.8 (1.2)	0.7 (0.7)	0.66
BNP, pg/ml	658.5 (940.4)	197.3 (181.1)	0.02^a^
NT-proBNP, pg/ml	7222.3 (9331.5)	1796.4 (3211.8)	0.00^a^
Baseline Cr, median(IQR)	93.20 (86.4,99.8)	93.05 (88.8,97.6)	0.72
Peak serum Creatinine, median(IQR)	259.8 (187.5,491.0)	197.3 (151.6,291.5)	0.00^a^
Serum creatinine increase, median (IQR), mmol/L	167.9(103.3,393.8)	106.9(56.5,196.5)	0.00^a^
**Maximal respiratory support, (*n*%)**			
HFNC	8 (8.8%)	12 (17.1%)	0.11
NPPV	8 (8.8%)	4 (5.7%)	0.46
IPPV	50 (54.9%)	38 (54.3%)	0.93
ECMO	25 (27.5%)	16 (22.9%)	0.51
Baro-trauma	4 (4.4%)	4 (5.7%)	0.73
**Severity of AKI, (*n*%)**			
Stage 1	14 (15.4%)	27 (38.6%)	0.00^a^
Stage 2	30 (33%)	20 (28.6%)	0.55
Stage 3	47 (51.6%)	23 (32.9%)	0.02^a^
**Nephrotoxic drug, (*n*%)**			
Vancomycin	6 (6.6%)	11 (15.7%)	0.06
Aminoglycoside	3 (3.3%)	12 (17.1%)	0.01^a^
Sulfamethoxazole- trimethoprim	24 (26.4%)	27 (38.6%)	0.09
Colistin	1 (1.1%)	12 (17.1%)	0.00^a^
Amphotericin B	5 (5.5%)	14 (20%)	0.01^a^
ACEI/ARB	1 (1.1%)	0 (0%)	1.00
NSAIDs	3 (3.3%)	12 (17.1%)	0.01^a^
Diuretics	17 (18.7%)	31 (44.3%)	0.00^a^
Voriconazole	11 (12.1%)	24 (34.3%)	0.00^a^
Cyclosporine	4 (4.4%)	0 (0%)	0.13
**Fluid balance**			
D1, median (IQR)	370 (–127,1451)	160 (–283,719.5)	0.07
D2, mean (*SD*)	270 (1677)	563.4 (1250.1)	0.23
D3, median (IQR)	–375 (–1373.8,678.5)	76 (–602,847)	0.06
D4, mean (*SD*)	–116.1 (1891.5)	132.5 (1323.1)	0.36
D5, mean (*SD*)	–67.5 (1962.9)	129.4 (1583.2)	0.51
D6, mean (*SD*)	196.7 (1322.9)	119.3 (1652.2)	0.76
D7, mean (*SD*)	–188.2 (1245.9)	489.7 (1566.9)	0.01^a^
CRRT, (*n*%)	28 (30.8%)	10 (14.3%)	0.02^a^
**Outcomes**			
Invasive ventilation-free days to day 28, mean (*SD*)	10.3 (11.7)	6 (9.1)	0.01^a^
ICU length of stay, mean (*SD*)	20.6 (19.5)	25.2 (23.1)	0.17
Hospital length of stay, mean (*SD*)	21.8 (24)	25.1 (29.9)	0.44
Renal function recovery within 28 days, (*n*%)	31 (34.1%)	23 (32.9%)	0.87
Mortality, (*n*%)	44 (48.4%)	48 (68.6%)	0.01^a^

^a^p < 0.05.

After adjusting for confounding factors, history of hypertension (*p* = 0.01), history of CKD (*p* = 0.03), and the SOFA score upon admission (*p* = 0.02) were significantly higher in the early-onset group than in the late-onset group. However, the use of nephrotoxic agents, especially colistin (*p* = 0.01), NSAIDs (*p* = 0.04) and diuretics (*p* = 0.03), was significantly associated with the development of late-onset AKI.

### Clinical outcomes of early and late acute kidney injury

In the subgroup analysis, both the ICU length of stay and hospital length of stay were shorter in the early AKI group, but the difference was not statistically significant. The number of invasive ventilation-free days to Day 28 was significantly longer and the mortality rate was lower in the early AKI group than in the late AKI group. However, in Kaplan–Meier analysis, the mortality rate between the two groups was not significantly different (*p* = 0.39).

## Discussion

We comprehensively discussed the effects of ARDS on renal function based on comorbid diseases, the etiology and severity of ARDS, hemodynamics, maximal respiratory support, fluid therapy and drug therapy.

In our study, AKI developed in half of the patients with ARDS during their ICU stay, and most of them developed AKI within the first 48 h after ARDS diagnosis. The risk factors for AKI in patients with ARDS were age, history of diabetes mellitus and CKD, admission APACHE II score, Lac level and the need for ECMO support. Patients with ARDS with AKI had a significantly higher 28-day mortality rate and shorter invasive ventilation-free days up to Day 28. Previous studies reported that the incidence of AKI in patients with ARDS ranged from 44.3 to 68.3% (8, 9) and that older age was associated with the development of AKI, which is in accordance with our study. We also found that DM increased the risk of AKI in patients with ARDS. Previous studies found that diabetes mellitus was the most commonly observed comorbidity in patients with AKI in both critically ill patients and non-critically ill patients (23–25). This comorbidity is an important cause of microvasculitis and macrovasculitis and may be associated with a higher susceptibility to ischemic–hypoxic insults, which predisposes patients to AKI (26). Moreover, we found that CKD was a significant risk factor for AKI. CKD has consistently been shown to be a significant risk factor for the development of AKI (27). Probable explanations include the hemodynamic instability and failure of autoregulation in CKD patients, the ease of detection of small changes in GFR when renal function is impaired, and a predisposition to further injury in patients with diminished renal function (28). The patients with AKI had higher admission APACHE II scores and higher levels of lactic acid, which suggests that these patients were admitted to the hospital with more serious illnesses, combined with multiple organ dysfunction and low organ perfusion. Our study also revealed that ECMO was a risk factor for AKI. The literature has reported that the incidence of AKI in patients on ECMO is as high as 70–85% (29, 30). The progression of pre-existing multisystemic disease, pre-ECMO management, activation of inflammatory mediators, alterations in renal macro/microvasculature, ischemia-reperfusion, hemolysis and oxidative stress, disruption of the glycocalyx, impaired renal autoregulation, and iatrogenic coagulation abnormalities are possible factors involved in the pathogenesis of ECMO-associated AKI (31).

We also observed that AKI significantly increased the mortality rate of ARDS. The in-hospital mortality was 57.1% in ARSD patients with AKI, which was significantly higher than the 42.4% mortality rate in patients with ARDS. Shock was the only independent risk factor for mortality in patients with AKI and patients with ARDS in our study. Previous studies’ results confirmed this conclusion. Huang et al. found that shock was independently significantly associated with hospital mortality in patients with ARDS (HR 2.017, 95% CI 1.308–3.111, *p* = 0.002) (21). In addition, concomitant shock may result in hypoperfusion and renal vasoconstriction, leading to acute tubular necrosis, which deteriorates renal function (32).

Moreover, we provided insight into the characteristics of AKI in patients with ARDS. We found that AKI occurring within 48 h of ARDS diagnosis appears to be strongly associated with the severity of primary disease and patient comorbidities, which may cause renal hypoxia and hypoperfusion, whereas late AKI may be more closely associated with drug toxicity (33). Therefore, during ARDS treatment, the application of nephrotoxic drugs should be avoided as much as possible. In addition, therapeutic drug monitoring uses drug concentrations and pharmacokinetic/pharmacodynamic objectives to manage a patient’s medication regimen, optimize outcome and prevent resistance or toxicity. We should routinely monitor therapeutic drugs during treatment.

Our study has several limitations. First, since most of the study population did not have a measured baseline serum creatinine, we addressed this problem using estimated serum creatinine by back calculation with the MDRD equation. Although this method has been widely used in previous studies, it still overestimates the incidence of AKI (8, 17, 34). To compensate for this problem, we defined AKI according to the KDIGO 2012 guidelines, which used both serum creatinine and urine volume. In addition, we incorporated confounding factors as comprehensively as possible and conducted multifactor analysis. Regarding the risk factors of early and late AKI, the application of some of the nephrotoxic drugs especially vancomycin, aminoglycoside and colistin usually occurred in the late stage of ARDS complicated by nosocomial infections. Therefore, late AKI has a greater chance of being exposed to this risk factor. This may introduce some bias. In addition, we did not follow up on patients’ renal function after discharge, so the entire clinical course of AKI in patients with ARDS could not be evaluated.

## Conclusion

The incidence of in-hospital AKI in patients with ARDS was 51.8% in our study. Age, a history of DM and CKD, initial severity of illness, lactic acid level and ECMO were associated with the development of AKI in ARDS patients. The mortality rate of ARDS patients combined with AKI was significantly higher than that of patients without AKI. Shock was the only risk factor for mortality in patients with ARDS with AKI. Furthermore, we found that early-onset AKI in patients with ARDS was associated with the severity of primary disease and patient comorbidities, whereas late-onset AKI was more closely associated with drug toxicity. Therefore, therapeutic drug monitoring should be routinely used to avoid drug toxicity during treatment.

## Data availability statement

The raw data supporting the conclusions of this article will be made available by the authors, without undue reservation.

## Ethics statement

The studies involving human participants were reviewed and approved by Ethics Committee of the China-Japan Friendship Hospital (the number of ethics board was 2015-77). The patients/participants provided their written informed consent to participate in this study.

## Author contributions

XC and XH designed the research project, analyzed the data, and drafted the manuscript. XC, XY, XH, YC, YT, and QZ performed the study and collected the clinical data. XC, XH, and QZ revised and approved the final version of the manuscript. All authors contributed to the article and approved the submitted version.

## Abbreviations

ARDS: acute respiratory distress syndrome
AKI: acute kidney injury
ICU: intensive care unit
ECMO: extracorporeal membrane oxygenation
KDIGO: kidney disease improving global outcomes
SCr: serum creatinine
MDRD: modification of diet in renal disease
GFR: glomerular filtration rate
CKD: chronic kidney disease
RRT: renal replacement treatment
APACHE II score: acute physiology and chronic health evaluation II score
SOFA score: sequential organ failure assessment
ACEIs/ARBs: angiotensin converting enzyme inhibitors/angiotensin receptor blockers
NSAIDs: non-steroid anti-inflammatory drugs
OR: odds ratio
CI: confidence interval.
